# Supplementary material for: Effects of a 16-Week Green Exercise Program on Body Composition, Sleep, and Nature Connection in Postmenopausal Women
Source: Int J Environ Res Public Health. 2025 Aug 1;22(8):1216. doi: 10.3390/ijerph22081216 (PMC12385796; doi:10.3390/ijerph22081216)
Supplement: Supplementary file 1 [file ijerph-22-01216-s001.zip › Supplementary Material S1.pdf]

Supplementary Material S1.

MENO(S)PAUSA+MOVIMENTO

PROGRAM FOR THE PROMOTION OF EXERCISE AND HEALTH IN POSTMENOPAUSAL WOMEN

INFORMED CONSENT

1. MENO(S)PAUSA+MOVIMENTO is a program for the promotion of exercise and health aimed at postmenopausal women residing in the municipality of Penafiel. It is promoted by the Penafiel City Council, in collaboration with the Regional Health Administration of the North, and under the scientific-pedagogical supervision and coordination of the University of Trás-os-Montes and Alto Douro.
2. This program aims to implement a set of free screenings for various parameters related to the health of postmenopausal women and to develop a physical activity program in contact with the natural environment, considering the physical and psychological changes resulting from menopause and aging. The results obtained will be communicated in writing to all program participants, with clarification provided, and will also be used in research to advance knowledge related to the prescription and monitoring of physical activity in this population. As women live approximately one-third of their lives post-menopause, play a key role in providing home and community care, and face greater gender-based health inequalities, improving women's health is important both to them and to society in general. Given the increasing number of people living in urban areas and the need to support physical activity participation, MENO(S)PAUSA+MOVIMENTO is also aimed at evaluating/classifying some of the pedestrian trails in the municipality, making them attractive for promoting a more active lifestyle among this population.
3. In this program, body fat, muscle mass, and bone condition will be assessed using bioimpedance and ultrasound. Cardiorespiratory fitness will be measured through a submaximal protocol, and handgrip strength will be used to assess muscular condition. Physical activity, as well as sleep duration and quality, will be measured using accelerometers (wristwatch-like devices). Cardiovascular and metabolic risk will be assessed via blood tests, arterial stiffness (loss of elasticity in artery walls), and heart rate variability. Quality of life, connection to the natural environment, and demographic data will be assessed through a questionnaire, while postural stability will be analyzed to assess fall risk.
4. All assessments will be carried out under professional supervision and will pose no risk to participants' safety. Preparation guidelines for some of these assessments will be provided in advance in writing.
5. Participation in MENO(S)PAUSA+MOVIMENTO may also include a multimodal exercise program (combining physical exercises with components such as cardiorespiratory fitness, muscular strength, and flexibility), conducted 3 times per week, emphasizing interaction with the natural environment.
6. Participants in the exercise program will be covered by personal accident insurance that will apply in cases where an accident/injury occurs within the scope of and as a result of this program, with clear causal connection.
7. Confidentiality of participants will be ensured, and data will be used exclusively for the program. It is guaranteed that participant identification will never be made public, and all contact will be made in a private setting. Data may be published, but participant identities will never be disclosed.
8. Participation in the program does not involve any compensation or payment; participation is voluntary.
9. Participants may withdraw their consent at any time and may object to the processing of their personal data collected for the MENO(S)PAUSA+MOVIMENTO program without any consequences, particularly regarding health care.
10. Personal data collected and processed for the MENO(S)PAUSA+MOVIMENTO program will be retained only for the period strictly necessary for the program's purposes, after which it will be permanently destroyed by those responsible for it.

11. Personal data collected and processed for the MENO(S)PAUSA+MOVIMENTO program will not be shared with third parties under any circumstances.

After being duly informed, if you wish to participate in the study, please mark with a cross the option below:

☐ I declare that I have read and understood this document, as well as the verbal information provided to me by the MENO(S)PAUSA+MOVIMENTO program coordinators. In this context, I agree to participate in the program and authorize the collection and processing of my data, with the guarantee of confidentiality and anonymity, in accordance with Regulation (EU) 2016/679 of the European Parliament and Council, of April 27, 2016 (General Data Protection Regulation – GDPR), in effect since May 25, 2018.

Participant's Signature: _________________________ Date: __/__/202_

We certify that the nature, objectives, and potential benefits of participation in the MENO(S)PAUSA+MOVIMENTO program have been explained to the above-mentioned participant, and we witnessed the signature above. This document consists of two pages, and a copy will be provided to the study participant.

____

Maria Helena Rodrigues Moreira
Associate Professor at UTAD
Scientific and pedagogical coordinator
of the MENO(S)PAUSA+MOVIMENTO

____

Maria Emília de Moura Alves
Technical-scientific and pedagogical coordinator of the
MENO(S)PAUSA+MOVIMENTO
