# Supplementary material for: Effects of a 16-Week Green Exercise Program on Body Composition, Sleep, and Nature Connection in Postmenopausal Women
Source: Int J Environ Res Public Health. 2025 Aug 1;22(8):1216. doi: 10.3390/ijerph22081216 (PMC12385796; doi:10.3390/ijerph22081216)
Supplement: Supplementary file 1 [file ijerph-22-01216-s001.zip › Supplementary Material S2.pdf]

Supplementary Material S2. Cardiorespiratory exercise progression during the 16-week intervention

**FREQUENCY, INTENSITY, TIME AND TYPE OF CARDIORESPIRATORY EXERCISE**																
**Week**	1°	2°	3°	4°	5°	6°	7°	8°	9°	10°	11°	12°	13°	14°	15°	16°
**Frequency**	3 times a week															
**Intensity**	30% – 40% of the HR reserve		40% – 60% of the HR reserve			50% – 70% of the HR reserve					60% – 75% of the HR reserve					
	9–12 BPS		12–14 BPS			13–15 BPS					14–16 BPS					
**Time**	20 minutes			25 minutes					30 minutes							
**Type**	Walk				Walk/Run											

HR – Heart rate; **BPS** - Borg perceived exertion scale (6–20)
