# Supplementary material for: Effects of a 16-Week Green Exercise Program on Body Composition, Sleep, and Nature Connection in Postmenopausal Women
Source: Int J Environ Res Public Health. 2025 Aug 1;22(8):1216. doi: 10.3390/ijerph22081216 (PMC12385796; doi:10.3390/ijerph22081216)
Supplement: Supplementary file 1 [file ijerph-22-01216-s001.zip › Supplementary Material S3.pdf]

Supplementary Material S3. Resistance exercise structure in the program Meno(s)Pausa+Movimento.

RESTISTANCE EXERCISE																
**Week**	1ª	2°	3°	4°	5°	6°	7°	8°	9°	10°	11°	12°	13°	14°	15°	16°
**Frequency**	2 days/week															
**Intensity/ Type**	MUSCLE STRENGTH<br>40-50% 1RM<br>10-12 BPS<br>8-12 repetitions<br>2 sets<br>Light-to-moderate speed	MUSCLE STRENGTH<br>50-60% 1RM<br>10-12 BPS<br>8-12 repetitions<br>2 sets<br>Light-to moderate speed	MUSCLE STRENGTH<br>60% 1RM<br>12 BPS<br>8-12 repetitions<br>2 sets<br>Light-to-moderate speed	MUSCLE STRENGTH<br>60-70% 1RM<br>12-14 BPS<br>8-12 repetitions<br>2 sets<br>Light-to-moderate speed	MUSCLE STRENGTH<br>70% 1RM<br>14 BPS<br>8-12 repetitions<br>2 sets<br>Light-to-moderate speed	MUSCLE STRENGTH<br>70% 1RM<br>14 BPS<br>8-12 repetitions<br>2 sets<br>Light-to-moderate speed	MUSCLE STRENGTH<br>70-80% 1RM<br>14-16 BPS<br>8-12 repetitions<br>2 sets<br>Light-to-moderate speed	MUSCLE STRENGTH<br>80% 1RM<br>16 BPS<br>8-12 repetitions<br>2 sets<br>Light-to-moderate speed	MUSCLE STRENGTH<br>80% 1RM<br>16 BPS<br>8-12 repetitions<br>2 sets<br>Light-to-moderate speed	MUSCLE POWER<br>40% 1RM<br>10 BPS<br>(upper body)<br>60% 1 RM<br>12 BPS<br>(lower body)<br>6-10 repetitions<br>Speed fast	MUSCLE STRENGTH<br>80% 1RM<br>16 BPS<br>8-12 repetitions<br>2 sets<br>Light-to-moderate speed	MUSCLE POWER<br>45% 1RM<br>11 BPS<br>(upper body)<br>60% 1 RM<br>12 BPS<br>(lower body)<br>6-10 repetitions<br>Speed fast	MUSCLE STRENGTH<br>80% 1RM<br>16 BPS<br>8-12 repetitions<br>2 sets<br>Light-to-moderate speed	MUSCLE POWER<br>45% 1RM<br>11 BPS<br>(upper body)<br>70% 1 RM<br>14 BPS<br>(lower body)<br>6-10 repetitions<br>Speed fast	MUSCLE STRENGTH<br>80% 1RM<br>16 BPS<br>8-12 repetitions<br>2 sets<br>Light-to-moderate speed	MUSCLE POWER<br>45% 1RM<br>11 BPS<br>(upper body)<br>70% 1 RM<br>14 BPS<br>(lower body)<br>6-10 repetitions<br>Speed fast
**Time**	20 minutes	20 minutes	20 minutes	15 minutes	15 minutes	15 minutes	15 minutes	15 minutes	10 minutes	10 minutes	10 minutes	10 minutes	10 minutes	10 minutes	10 minutes	10 minutes
**Some notes**	• Work on the 4 pillars of movement: locomotion, change of level, push-pull and rotation;<br>• Working agonist and antagonist muscles in the same session;<br>• Performing polyarticular exercises before monoarticular ones;<br>• Stimulating large muscle groups and then small muscle groups;<br>• Performing concentric, eccentric and isometric contractions;<br>• Emphasising large muscle groups (arms, chest, abdominals, lower limbs and back) and areas of the body that are more sensitive to sarcopenia and osteoporosis;<br>• Working the leg extensors (quadriceps), leg flexors (semitendinosus, semimembranosus, biceps femoris, gastrocnemius, plantaris, popliteus, rectus internus, hamstrings), dorsiflexors (tibialis anterior, extensor digitorum propria, extensor digitorum communis, peroneus anterioris) and plantar flexors (triceps suralis, longus peroneus lateralis, tibialis posterioris, flexor digitorum profundus, flexor digitorum communis), which are important for improving balance, mobility and the risk of falls.<br>• Consider performing isometric and dynamic exercises in the trunk region, due to their benefits in terms of speed, cadence and stride length;<br>• Work on the extensor muscles of the trunk (trapezius and muscles of the common mass), posterior thigh, glutes, abdominals (prioritise isometric work) and scapular retraction and depression exercises in situations of osteoporosis of the spine.																
**Myofascial meridians**	Anterior superficial line<br>Posterior superficial line	Functional lines<br>Deep line frontal	Anterior superficial line<br>Posterior superficial line	Spiral thread<br>Functional lines	Anterior superficial line<br>Posterior superficial line	Sideline<br>Line arm Line	Anterior superficial line<br>Posterior superficial line	Functional lines<br>Deep line frontal	Anterior superficial line<br>Posterior superficial line	Spiral thread<br>Functional lines	Anterior superficial line<br>Posterior superficial line	Sideline<br>Line arm Line	Anterior superficial line<br>Posterior superficial line	Functional lines<br>Deep front line	Anterior superficial line<br>Posterior superficial line	Spiral thread<br>Functional lines

1 RM – 1 maximum repetition; **BPS** - Borg perceived exertion scale (6-20)
